# Control of unreasonable growth of medical expenses in public hospitals in Shanghai, China: a multi-agent system model

**DOI:** 10.1186/s12913-020-05309-z

**Published:** 2020-06-03

**Authors:** Wenya Yu, Xiang Liu, Fangjie Zhao, Meina Li, Lulu Zhang

**Affiliations:** 1 School of Public Health, Shanghai Jiao Tong University School of Medicine, Shanghai, 200025 China; 2 Department of Military Health Service Management, College of Military Health Service Management, Second Military Medical University, Shanghai, 200433 China; 3 Department of Respiratory Disease, The 903rd Hospital of PLA, Hangzhou, 310000 Zhejiang China

**Keywords:** China, Health policy, Healthcare reform, Model, Multi-agent system, Public hospital, Shanghai

## Abstract

**Background:**

This study aims to establish a multi-agent system model to provide accurate suggestions for the policy proposal of controlling the unreasonable growth of medical expenses charged by public hospitals in China.

**Methods:**

A multi-agent system model was employed in this study. Agents of this model were divided into patients, doctors, medical institutions, the government, and medical insurance agencies. The model was composed of two subsystems: the disease and medical-seeking subsystem, and the medical expenses subsystem. Policy intervention experiments were conducted on patients’ medical-seeking preferences, doctors’ public welfare behaviors, and the government’s financial investment.

**Results:**

At present, medical expenses in China are unreasonable and keep increasing, and the proportion of medicine and physical examination expenses to total medical expenses for public hospitals is unreasonable. Intervention experiments suggested that expanding the promotion and application of the community first-visit system could rationalize patients’ medical-seeking preferences, increasing doctors’ incomes and reducing workload could significantly restrict doctors’ over-prescription behaviors. Also, improving the government’s financial investment could guide public hospitals to strengthen their commitment to public welfare responsibilities. These interventions could decrease the unreasonable growth of medical expenses of public hospitals. The combined intervention effects on suppliers, demanders, and the government were better than the effect of these agents independently.

**Conclusions:**

The main reasons for the unreasonable increase in patient medical expenses at public hospitals could be attributed to patients’ unreasonable medical-seeking preferences, doctors’ weak public welfare incentives, and the government’s inadequate financial investment. Policy-makers should consider proposals to restrict and guide the behaviors of suppliers, demanders, and the government, simultaneously. The government should consider the feasibility, response speed, and implementation cost of policies as well.

## Background

Since the reform of public hospitals in China in 2009, the issue of unreasonable growth in medical expenses has gradually become the focus of reform. The Chinese government issued the “Notice on Certain Opinions on Controlling Unreasonable Growth of Medical Expenses in Public Hospitals” [1], clearly noting that it is necessary to control the unreasonable growth of medical expenses in public hospitals, especially the excessive increase of medical expenses in urban public hospitals. Considering public hospitals in Shanghai as an example, statistics [2–7] showed that the annual growth rates of both outpatient and inpatient medical expenses from 2011 to 2016 remained above 4% and even exceeded 6% in the last 2 years (Table 1). This suggests an unreasonable growth of medical expenses, especially when compared with the annual growth rates in the United states of 4.3% and the Organization for Economic Co-operation and Development (OECD) of 3.8% [8].

**Table 1:** Outpatient and inpatient medical expenses per capita and the annual growth rates in public hospitals of Shanghai, China (2011–2016)

Year	Outpatient medical expenses per capita (CNY^a^)	Annual growth rate of outpatient medical expenses (%)	Inpatient medical expenses per capita (CNY^a^)	Annual growth rate of inpatient medical expenses (%)
2011	252.8	NA	12,897.7	NA
2012	265.9	5.2	13,498.2	4.7
2013	276.0	4.1	14,243.2	5.5
2014	289.0	4.4	14,862.2	4.3
2015	306.9	6.2	15,935.7	7.2
2016	330.2	7.6	16,942.5	6.3

^a^*CNY* Chinese Yuan (currency unit)

The problem of unreasonable growth in medical expenses is related to the behaviors of multiple agents, including the government, suppliers, and demanders. Studies in OECD countries and the United States also demonstrated that factors that influence healthcare expenditure are complicated an span all aspects of the health system, such as prices, volumes, supply, demand, and market processes [9–11]. In China, specifically, there are several issues that are likely contributors to the growth in medical expenses. First, the 2010–2016 financial subsidy income accounted for only 7.71–9.13% of the total revenue of public hospitals [7], which means that public hospitals’ revenue came mainly from their operating income. This unreasonable financial revenue structure has led to profit-seeking by public hospitals, as well as the emergence of “economic man” behaviors [12] such as over-prescribing, inducing over-medication, and conducting unnecessary physical examinations [13–16], which eventually caused an unreasonable increase in medical expenses. Second, as the main body of medical service suppliers, medical staff could not realize their own technology and labor value because of the inadequate financial support and salary system. This aggravated the profit-seeking behavior of medical personnel [17, 18]. Third, patients’ unreasonable medical-seeking behaviors caused an unnecessary increase in medical expenses [19]. To understand the medical-seeking behaviors of patients, it is necessary to understand the three-level healthcare system in China. This system consists of primary healthcare institutions (e.g., community healthcare centers and rural healthcare clinics), secondary hospitals (e.g., county or district hospitals), and tertiary hospitals (e.g., topmost hospitals). However, the Chinese healthcare system does not involve a strict general practitioner (GP) and referral system. Therefore, patients in China can enjoy considerable freedom to choose to attend any hospital based on their personal preferences [19, 20]. For example, patients with minor illnesses, common diseases, and chronic conditions can always choose the topmost hospitals and fail to be diverted to more appropriate primary medical institutions.

Controlling the unreasonable growth of medical expenses is therefore key to China’s healthcare reform. Many scholars have explored the unreasonable growth of medical expenses with the majority focused on the medical expenses of a certain disease and its influencing factors [21–24]. Others explored strategies of controlling unreasonable growth of medical expenses from the perspectives of a supervision system, clinical pathways, supplier behaviors, compensation mechanisms, and medicine market management [25–27]. A few studies focus on the effects of medical insurance on controlling medical expenses [28–30]. These studies were primarily conducted from the perspective of a single agent through quantitative or qualitative methods, and proposed limited suggestions or explored some influencing factors rather than predicting the effects of the controlling proposals. However, this issue is related to the behaviors of multiple agents, and the problem of unreasonable growth in medical expenses in public hospitals is a typical multi-agent system (MAS) problem. MAS consists of multiple agents, with interactions among agents, and between agents and environments, which together constitute the overall performance of the MAS [31]. The behaviors of these agents are highly complex. Different agents will evaluate based on their own characteristics, the interaction of their own behaviors with those of other agents, and the interaction between their own behaviors and the environment. Further, they make adaptively behavioral changes, which are reflected externally in the changes of medical expenses in public hospitals.

Therefore, MAS modeling is an appropriate method to explore how the unreasonable growth of medical expenses are controlled in public hospitals, which enables the prediction of proposal results by implementing intervention experiments for relevant policies. The purpose of the study is to use MAS modeling to explore how the agents influence medical expenses and, based on the results, to provide suggestions for controlling the unreasonable growth of medical expenses, and to improve the accuracy of policies.

## Methods

### Model description

#### Problem

The unreasonable medical-seeking behavior of patients [19], excessive pursuit of profits caused by the unreasonable salary system of medical staff [17, 18, 32, 33], and insufficient government financial subsidies in public hospitals [7, 12] all have a direct or indirect effect on medical expenses. The control of unreasonable increases in medical expenses must consider the behaviors and impact mechanisms of these agents.

#### Agents and associations

The model includes five categories of agents: patients, doctors, medical institutions, the government, and medical insurance agencies. Patient agents are randomly generated based on the characteristics and patterns of various diseases. According to the patients’ medical-seeking preferences [19], together with consideration of the characteristics, level, and geographical location of the medical institutions, and the reimbursement proportion of the medical insurance agency, patients with different characteristics will be matched with their preferred medical institutions, which connects the patient and medical institution agents. After the patients enter medical institutions for treatment, the behavioral characteristics of doctors will affect the medical services that are accepted by the patients. The behaviors of the doctors are affected by the salaries and value orientation of the medical institution which, in turn, depends on the policy orientation and financial investment of the government agent. When faced with the diagnosis, prescription, and medical advice of doctors, whether patients will comply with their doctors’ advice is affected by their economic status, which usually depends on the reimbursement proportion of the medical insurance agency.

**Fig. 1:**
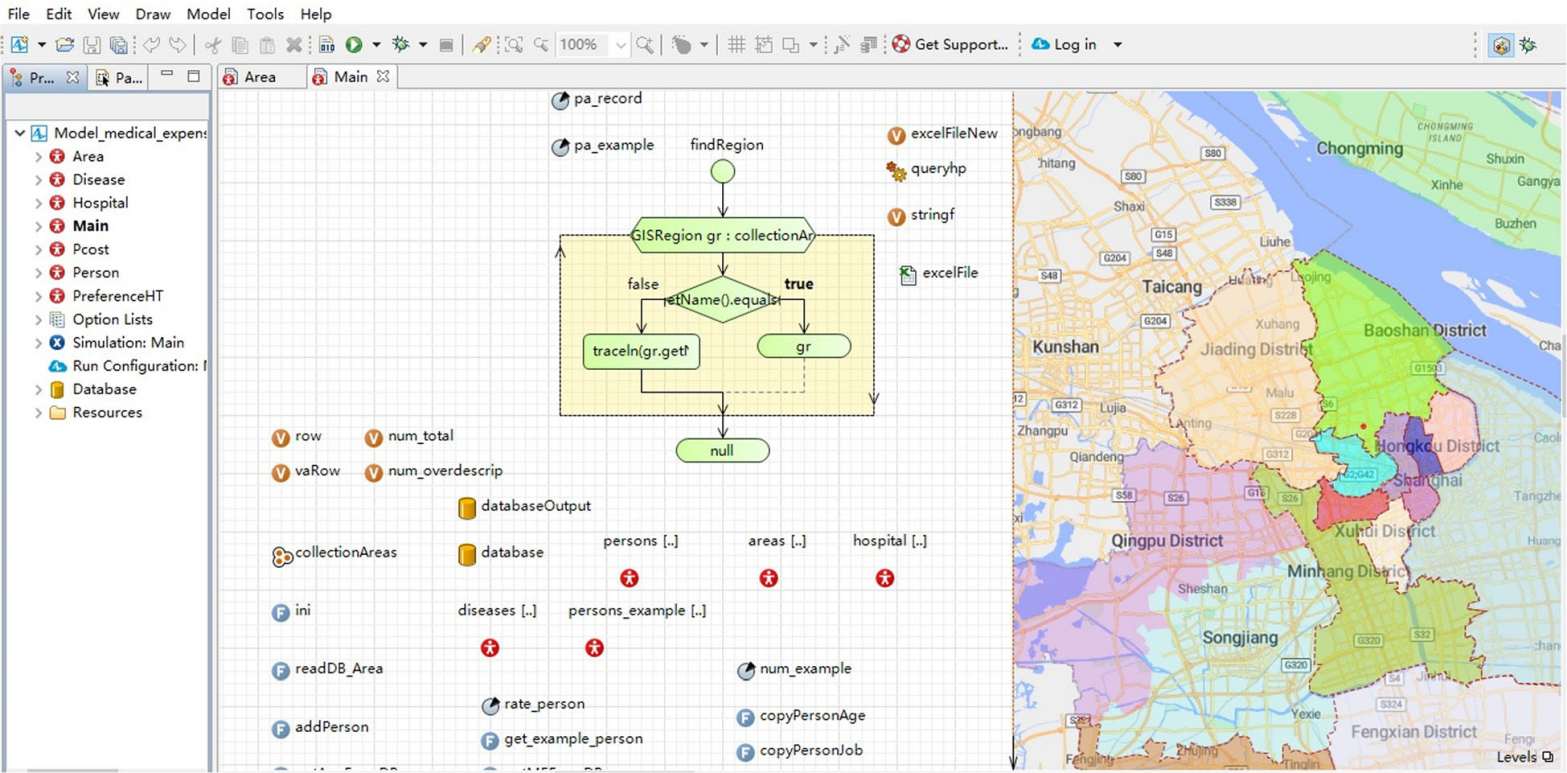
Multi-agent system model of controlling unreasonable growth of medical expenses in public hospitals

In addition to the specific characteristics of diseases, medical expenses are affected by the preferences of patients for the type of medical institution, behavior of doctors, policy orientation of the government, and reimbursement by the medical insurance agency. Jointly, these five types of agents determine medical expenses through their own characteristics and their mutual associations. The model was built by AnyLogic software (AnyLogic 8.2.4 Professional) (Fig. 1).

### Hypothesis of model

This model is used to simulate how medical expenses are generated. Based on the law of objective facts, and considering the simplification and abstraction of the model, this study carried out the following necessary logical assumptions that respect objective law (Table S2):

(1) Because there are many different types of diseases, considering the comprehensive coverage of all diseases and for reasons of simplification, this study classified diseases into 30 types based on national statistics. Patients were identified according to two-week prevalence rates [34, 35] of 30 diseases, which were derived from the *China Health and Family Planning Statistical Yearbook*. Patients with different diseases were generated randomly by computer. However, due to complexity and considering that this model does not focus on disease transmission, this study assumed that all populations are distributed statically according to Shanghai’s population density in different administrative districts, regardless of the population’s mobility and the impacts of disease transmission.

(2) Many doctors’ behaviors affect medical expenses and one of the most important is over-prescription [15]. To quantify and simplify the public welfare behavior of doctors, the model assumed that the over-prescription behavior of doctors is a key factor affecting their public welfare behavior, which is influenced by both their incomes and workload [15, 36]. In addition, the Chinese doctors’ salary is composed with a fixed salary and bonus, and the fixed salary is from the government, which means that doctors in different types of hospitals enjoy a similar fixed salary and its growth rate [37]. Furthermore, because this model is established based on the health delivery system in Shanghai, it is understandable to assume that the growth rate of bonus in different types of hospitals as the same in a certain area. Therefore, considering such two reasons and the lack of reported data [38], the annual growth rates for doctors’ incomes in different types of hospitals were assumed to be the same and was extracted from a previous national research study [39].

(3) Though the effect was not obvious, previous publications have demonstrated that the development of primary healthcare institutions could affect patients’ medical-seeking preferences and reduce medical expenses. Moreover, that the Chinese government is making great efforts to support the community first-visit system and is investing heavily in primary healthcare institutions [40, 41]. Therefore, this model assumed that the government’s encouragement of the community first-visit system will affect a patient’s medical-seeking preference. In addition, the government’s financial subsidy for public hospitals influences medical expenses.

(4) Considering that this study aims to discuss the rationalization of medical expenses in public hospitals from the perspective of behaviors of the government, suppliers, and demanders, the model assumed that the effect of medical insurance agencies on patients’ medical-seeking preferences and medical expenses was solely achieved through the reimbursement proportion of medical insurance. The effect of the reimbursement proportion of medical insurance was realized by different proportions in different levels of healthcare institutions [42]. For example, if patients choose primary healthcare institutions, they only have to pay for 30% of the total medical expenses; if they choose tertiary hospitals, the personal proportion accounts for 50%. Therefore, if a patient was suffering from a mild illness (e.g., a cold) and this person could get equal quality services from all healthcare institutions, he / she would choose primary healthcare institutions because of the lower out-of-pocket expense.

### Subsystems of the model

Based on the logic of generating medical expenses, the MAS model developed for controlling the unreasonable growth of medical expenses in public hospitals contained two subsystems: the disease and medical-seeking subsystem, and the medical expenses subsystem.

#### Disease and medical-seeking subsystem

**Fig. 2:**
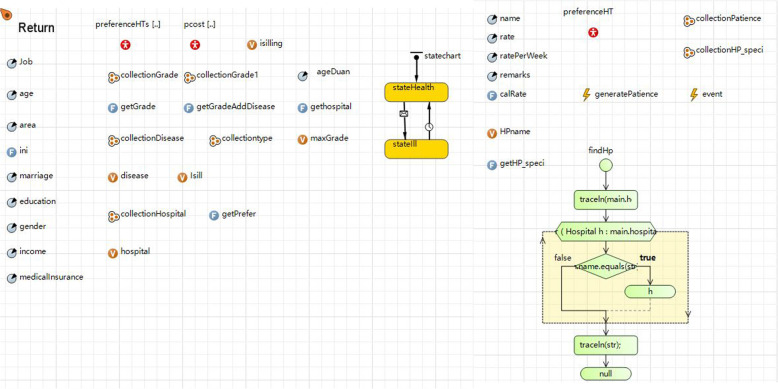
Disease and medical-seeking subsystem

As shown in Fig. 2, the disease and medical-seeking subsystem simulated the generation of the patient agents (starting point of the model) and the medical-seeking process. According to the two-week prevalence rates of 30 diseases noted above [7], patients were randomly generated by computer. Demographic and socioeconomic characteristics of the simulated patients included gender, age, occupation, marital status, education level, and income. The proportion of patients with different genders, ages, occupations, marital status, education levels, incomes and medical insurances were generated according to the actual distribution of the total population in Shanghai. Data were derived from government statistical reports and unpublished surveys [7, 43]. The simulated patients were distributed in different locations and the population densities of different locations were based on the actual population density of all 16 administrative districts of Shanghai [43]. Every patient had their own site (located by latitude and longitude). Patients with different characteristics had different medical-seeking preferences [19]; therefore, every patient was assigned their own basic preference according to their demographic characteristics and diseases. At this stage, the medical-seeking preferences were choices for healthcare institution levels (primary, secondary, and tertiary) and categories (comprehensive and specialized). However, if a patient had several choices without any preferences at the first stage, the model calculated the distances from this person to hospitals (every hospital was located by the actual latitude and longitude) around him / her. The simulated patient was then assigned the closest hospital. In summary, each simulated patient had their own preference for a hospital type at the first stage according to the rules mentioned above.

In addition, in this subsystem, government initiatives influenced patients’ medical-seeking preferences as well. The government agent guided patients with common diseases or minor illnesses to visit community health service centers first, through policies such as the community first-visit system, dual-referral system, and family doctor contracting system. However, during this stage, these initiatives had not played an obvious role in shifting the flow of patients from high-level institutions to primary healthcare institutions [40]. Therefore, the effects of these policies and whether they would encourage more patients to choose primary healthcare institutions was simulated and discussed in the policy intervention experiments.

#### Medical expenses subsystem

**Fig. 3:**
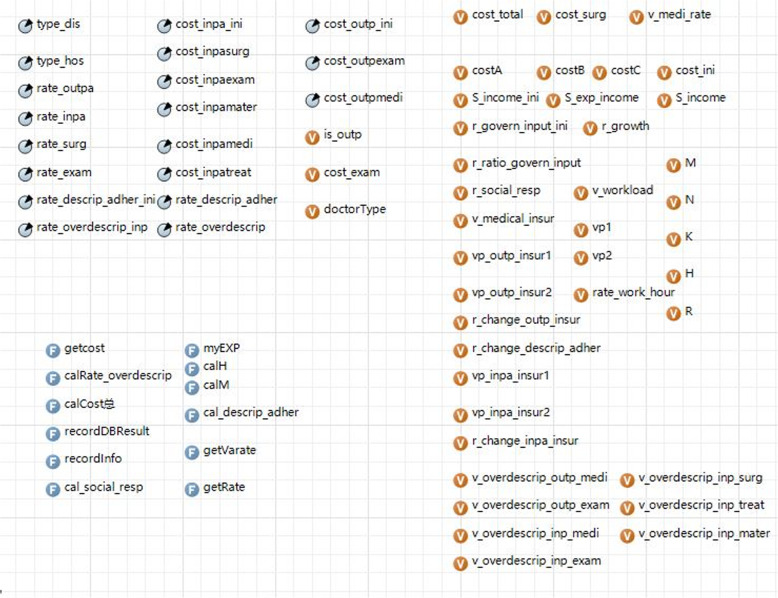
Medical expenses subsystem

As shown in Fig. 3, the medical expenses subsystem simulated the process of generating medical expenses in public hospitals. When patients selected a medical institution, their medical expenses were randomly generated by computer according to basic medical expenses and the diagnosis and treatment plans of various diseases in different medical institutions. We invited physicians to organize expert consultations to discuss and determine the probabilities of outpatient visits, hospitalization, surgery, and physical examinations for 30 diseases. Because we lacked the exact values of medical expenses, we calculated all related expenses according to the statistical values reported by the national health statistical yearbook [7], including the outpatient and inpatient basic medical expenses, medicine expenses, physical examination expenses, treatment expenses, surgery expenses, and medical material for 30 diseases in different levels of healthcare institutions. Considering the economic rules, this model also calculated the growth of medical expenses over time combined with the annual growth rates of different expenses, which were also calculated based on data from the national health statistical yearbook [7]. In this way, we determined the preliminary medical expenses.

However, the final medical expenses of each patient were influenced by many other factors. First, over-prescription by doctors increased the medical expenses. Whether doctors over-prescribed was determined by the gap between their actual and expected incomes, and the probabilities and degree of over-prescription in different situations (if expected incomes ≥ actual incomes, 10% of doctors would over-prescribe; otherwise, 50% of doctors would over-prescribe), as calculated from the expert consultations. The actual income of a doctor was determined by their professional title, as reported by publications and the Chinese physicians White Book [44–46], while the expected income was determined by workload (expected income = − 12,685.5 + 678.13 * working hours) [36]. Both actual and expected incomes changed over time. Second, the financial investment from the government agent affected the commitment to social responsibilities by medical institutions, which would then influence medical expenses by discouraging doctors from over-prescribing [40]. The more social responsibilities undertaken by healthcare institutions, the lower the probabilities of over-prescription were. Third, not all patients would accept all prescriptions; their prescription adherence influenced the final medical expenses. For example, if a doctor prescribed two drugs, a patient potentially only bought one. Some studies found that economic factors, especially medical insurance, were one of the most important factors of prescription adherence [47, 48]. Therefore, this model included the reimbursement proportions of patients’ medical insurance as the main measurement of prescription adherence. In summary, the final medical expenses were calculated based on the preliminary values, doctors’ over-prescription odds, government investment, commitment to social responsibilities, and patients’ prescription adherence.

### Variables and equations

The initial values of the input variables were derived from the *China Health and Family Planning Statistical Yearbook*, “Shanghai 2010 National Census Main Data Bulletin,” the “China Physician White Paper”; field and literature research; and expert consultations. The output variables of the model included different medical expense values. Initial input values of parameters are listed in Table S1.

#### Variables related to the patient agent

These included the total population, population distribution, two-week prevalence rates of 30 different diseases gender, age, occupation, marital status, income, education level, medical-seeking preferences, and adherence rate of prescriptions.

#### Variables related to the medical institution agent

These included the number, name, category, and geographical location (latitude and longitude) of tertiary comprehensive hospitals, specialized hospitals, district hospitals, and community health service centers.

#### Variables related to the doctor agent

These included the doctor’s professional title, income, annual growth rate of income, weekly hours worked, annual change rate for weekly hours worked, and the probability of over-prescription.

#### Variables related to the government agent

These included the proportion of the government’s financial subsidies to public hospitals and the promotion degree of the community first-visit system.

#### Variables related to the medical insurance agency agent

These included the composition of basic medical insurance for urban workers, basic medical insurance for urban and rural residents, and proportion of non-medical insurance residents.

#### Variables related to medical expenses

These included basic outpatient medical, medicine, and physical examination expenses; and basic inpatient medical, medicine, physical examination, treatment, surgery expenses, and medical material expenses for 30 diseases in four types of medical institutions; annual growth rate of different expenses; and probabilities of outpatient visits, hospitalization, surgery, and physical examinations.

### Model validation

The model validation was tested by comparing the simulated and real values. If the difference is between − 10 and 10%, the model can be considered to be correct and credible [34]. In the validation test for this model, the initial values of parameters were from 2014 data, and the model was set to run for 2 years. Therefore, the simulated medical expenses of 2015 and 2016 were obtained. These simulated medical expenses were compared with actual medical expenses in Shanghai derived from the *China Health and Family Planning Statistical Yearbook*.

The results showed that the gap between the simulated and actual values was between − 10 and 10%. The model was thus correct and credible (Table 2).

**Table 2:** Model validation

	2015			2016		
	Simulated value	Actual value	Difference	Simulated value	Actual value	Difference
Outpatient total medical expenses (CNY^a^)	304.2	306.9	−0.9%	311.0	330.2	−5.8%
Outpatient medicine expenses (CNY^a^)	176.5	168.5	4.7%	178.1	173.0	2.9%
Outpatient physical examination expenses (CNY^a^)	35.6	34.4	3.5%	36.4	39.0	−6.7%
Ratio of outpatient medicine to total medical expenses	58.0%	54.9%	5.6%	57.3%	52.4%	9.4%
Ratio of outpatient physical examinations to total medical expenses	11.7%	11.2%	4.5%	11.7%	11.8%	−0.8%
Inpatient total medical expenses (CNY^a^)	15,789.0	15,935.7	−0.9%	15,487.4	16,942.5	−8.6%
Inpatient medicine expenses (CNY^a^)	5382.1	5523.7	−2.6%	5166.4	5584.0	−7.5%
Inpatient physical examination expenses (CNY^a^)	1005.3	933.9	7.6%	1019.1	1033.1	−1.4%
Ratio of inpatient medicine to total medical expenses	37.0%	34.1%	1.7%	33.4%	33.0%	1.2%
Ratio of inpatient physical examination to total medical expenses	6.4%	5.9%	8.5%	6.6%	6.1%	8.2%

^a^*CNY* Chinese Yuan (currency unit)

### Intervention scenarios

This study carried out intervention experiments on patients’ medical-seeking behaviors, doctors’ public welfare behaviors, and the government’s financial investment in public hospitals. The control group was the baseline situation without any intervention, and the simulation time was 3 years.

#### Policy intervention scenario for patients

##### Test 1

Degree of promotion and application of the community first-visit system was reduced by 50%, and patients preferred tertiary comprehensive hospitals.

##### Test 2

Degree of promotion and application of the community first-visit system increased by 50%, and patients’ preferences were rationalized.

##### Test 3

Degree of promotion and application of the community first-visit system increased by 100%, and patients’ preferences were rationalized.

#### Policy intervention scenario for doctors

##### Test 1

Doctors’ income decreased by 50%, income growth rate decreased by 50%, weekly working hours were maximized (60 h), and growth rate of hours worked weekly increased by 50%.

##### Test 2

Doctors’ income increased by 100%, income growth rate increased by 50%, weekly working hours decreased by 25%, and growth rate of hours worked weekly decreased by 50%.

##### Test 3

Doctors’ income increased by 200%, income growth rate increased by 100%, weekly working hours decreased by 50%, and growth rate of hours worked weekly decreased by 80%.

#### Policy intervention scenario for government

##### Test 1

Annual growth rate of financial investment for tertiary comprehensive, specialized, and district hospitals, and the rate for community health service centers decreased by 50%.

##### Test 2

Annual growth rate of financial investment for tertiary comprehensive, specialized, and district hospitals increased by 100%, and the rate for community health service centers increased by 75%.

##### Test 3

Annual growth rate of financial investment for tertiary comprehensive, specialized, and district hospitals increased by 200%, and the rate for community health service centers increased by 100%.

#### Combined policy intervention scenario

Based on the independent intervention experiments on patients, doctors, and the government (the three group experiments mentioned above), a combination of thresholds for each group of experiments was performed to intervene in the behaviors of patients, doctors, and the government, simultaneously.

## Results

### Baseline

Within the three-year simulation, the outpatient medical, medicine, and physical examination expenses all showed a small increasing trend. The ratio of outpatient medicine to total medical expenses decreased slightly (almost 60%), while the ratio of outpatient physical examination expenses increased slightly. The inpatient medical and medicine expenses, and the ratio of medicine to total medical expenses showed a downward trend; however, inpatient physical examination expenses and their ratio to total inpatient medical expenses both increased.

Most government investment was in community health service centers. Government financial subsidies for tertiary comprehensive public and specialized hospitals showed a small downward trend, while financial subsidies in district hospitals and community health service centers showed an increasing trend.

Patients’ medical-seeking preferences for the four types of public medical institutions did not show obvious changes. Patients who preferred district and tertiary comprehensive hospitals accounted for the largest percentage.

The probability of over-prescription fluctuated slightly. About half of doctors over-prescribed. The increase in medicine and physical examination expenses caused by doctors’ over-prescriptions showed a gradual downward trend (Table 3).

**Table 3:** Simulation results—baseline

Items		Year 1	Year 2	Year 3
Outpatient medical expenses	Total medical expenses (CNY)	304.2	311.0	314.5
	Medicine expenses (CNY)	176.5	178.1	176.1
	Physical examination expenses (CNY)	35.6	36.4	37.4
	Ratio of medicine to total medical expenses	58.0%	57.3%	56.0%
	Ratio of physical examinations to total medical expenses	11.7%	11.7%	11.9%
Inpatient medical expenses	Total medical expenses (CNY)	15,789.0	15,487.4	14,662.1
	Medicine expenses (CNY)	5382.1	5166.4	4753.9
	Physical examination expenses (CNY)	1005.3	1019.1	1033.8
	Ratio of medicine to total medical expenses	37.0%	33.4%	32.4%
	Ratio of physical examinations to total medical expenses	6.4%	6.6%	7.1%
Proportion of government’s financial subsidies to total income	Tertiary comprehensive hospital	6.2%	6.2%	6.2%
	Specialized hospital	9.3%	9.2%	9.1%
	District hospital	9.7%	9.7%	9.7%
	Community health service center	15.2%	15.6%	16.3%
Proportion of patients in a certain type of medical institution to total number of patients	Tertiary comprehensive hospital	40.2%	40.2%	40.2%
	Specialized hospital	1.9%	2.0%	1.9%
	District hospital	44.0%	44.0%	44.0%
	Community health service center	13.8%	13.9%	14.0%
Over-prescription	Probability of over-prescription	50.0%	50.2%	50.0%

### Policy intervention experiments on patient behavior

**Fig. 4:**
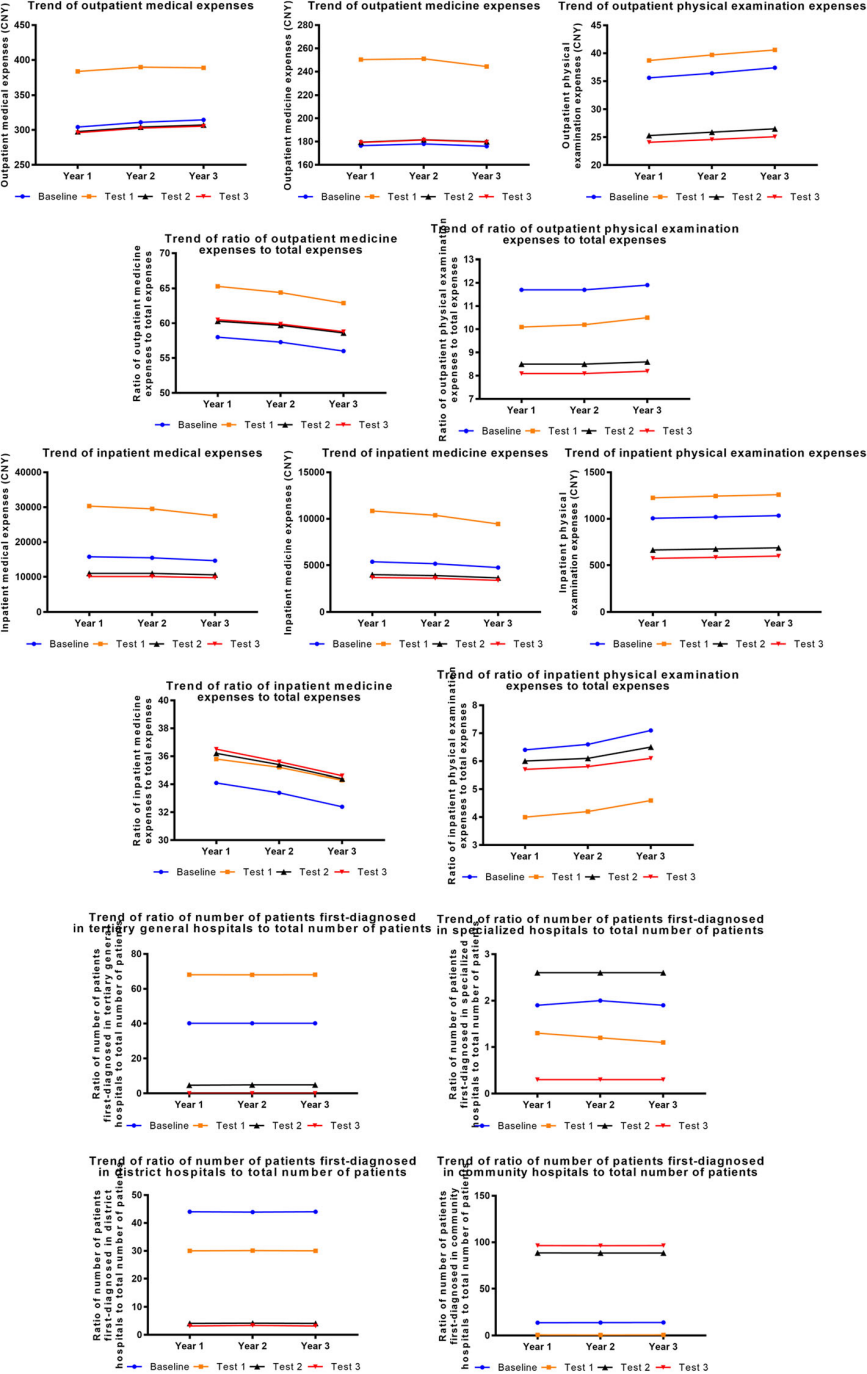
Results of policy intervention experiments on patient behaviors

Expanding the promotion and application of the community first-visit system could effectively control total medical, physical examination expenses, and their proportions. However, there was no effect on controlling medicine expenses and their proportions. When the promotion and application of the community first-visit system was expanded, more patients were guided to visit community health service centers and specialized hospitals first. More patients would, on the contrary, prefer tertiary comprehensive hospitals. However, when the degree of promotion and application of the community first-visit system increased to 1.5 times the current level, the effect of continuing to increase promotion and application on controlling medical expenses and optimizing patients’ medical-seeking preferences was not obvious, which indicated a marginal value (Fig. 4).

### Policy intervention experiments on doctor behavior

**Fig. 5:**
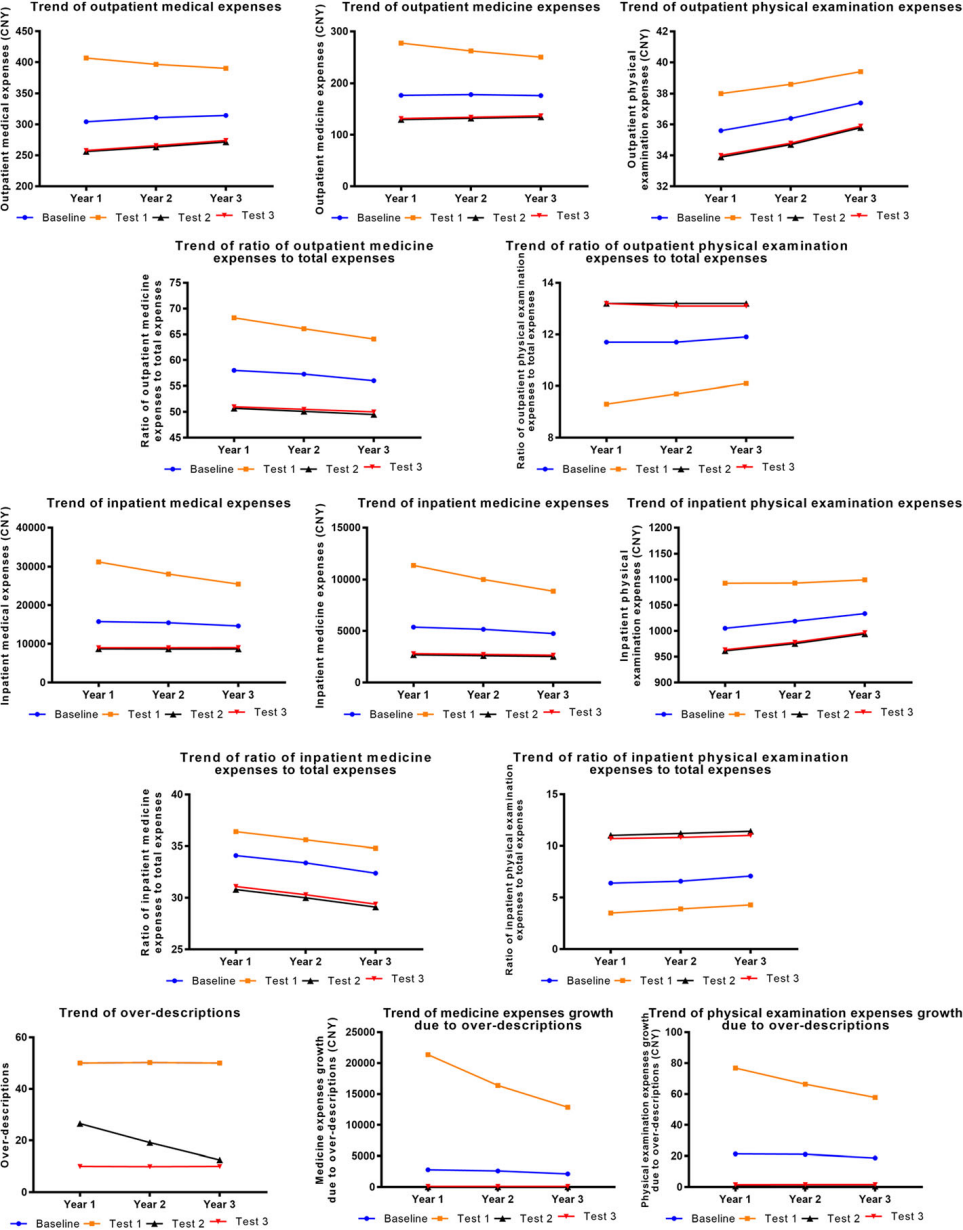
Results of policy intervention experiments on doctor behaviors

The adjustment of doctors’ income and workloads were two independent interventions. By simultaneously increasing doctors’ income and reducing their workloads, it could effectively control total medical expenses, medicine expenses and their proportion, and physical examination expenses; however, regulating the proportion of physical examination expenses was invalid. Moreover, this could weaken doctors’ incentives for over-prescription. It is worth noting that, when doctors’ income increased twofold and their working time decreased to 75% of the current level, marginal values appeared (Fig. 5).

### Policy intervention experiments on government behavior

**Fig. 6:**
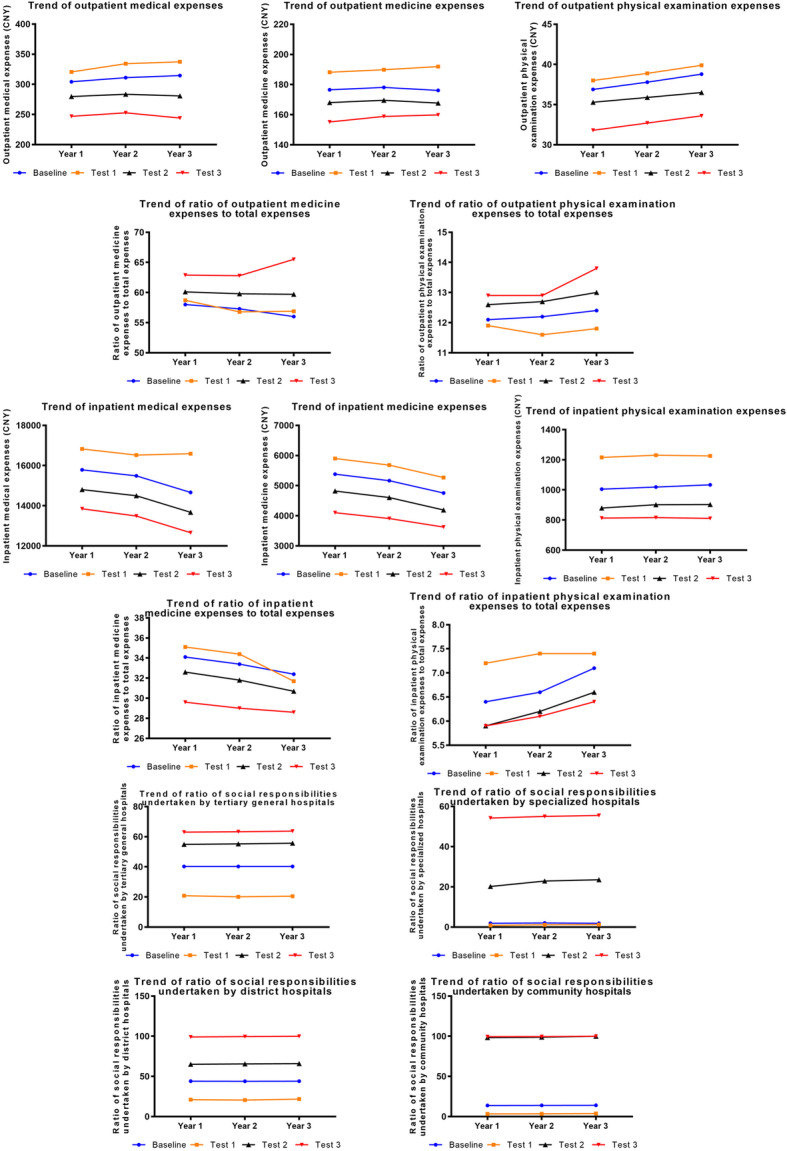
Results of policy intervention experiments on government behaviors

Increasing the government’s financial investment to medical institutions could effectively reduce total medical expenses, medicine expenses, proportion of inpatient medicine to total expenses, physical examination expenses, and proportion of inpatient physical examinations to total expenses, while regulating the proportion of outpatient medicine and physical examination expenses was not obvious. Moreover, the greater the proportion of the government’s financial investment, by encouraging medical institutions to undertake public welfare responsibilities, the more effective the effect on controlling medical expenses would be. However, when the government’s financial investment to community health service centers increased to 175% of the current degree, public welfare responsibilities would be undertaken perfectly, which suggests that the government’s financial investment in community health service centers should not exceed this level (Fig. 6).

### Combined policy intervention experiments

Based on the marginal values of the above three groups of experiments, the combined experimental interventions would be on patients, doctors, and the government, simultaneously. The scenario was the degree of promotion and application of the community first-visit system increased by 50% and patients’ preferences were rationalized; the doctor’s income increased by 100%, the growth rate of income increased by 50%, weekly working hours decreased by 25%, and the growth rate of weekly working hours decreased by 50%; the annual growth rate of financial investment for tertiary comprehensive, specialized, and district hospitals increased by 100%, and the annual growth rate of financial investment for community health service centers increased by 75%.

**Fig. 7:**
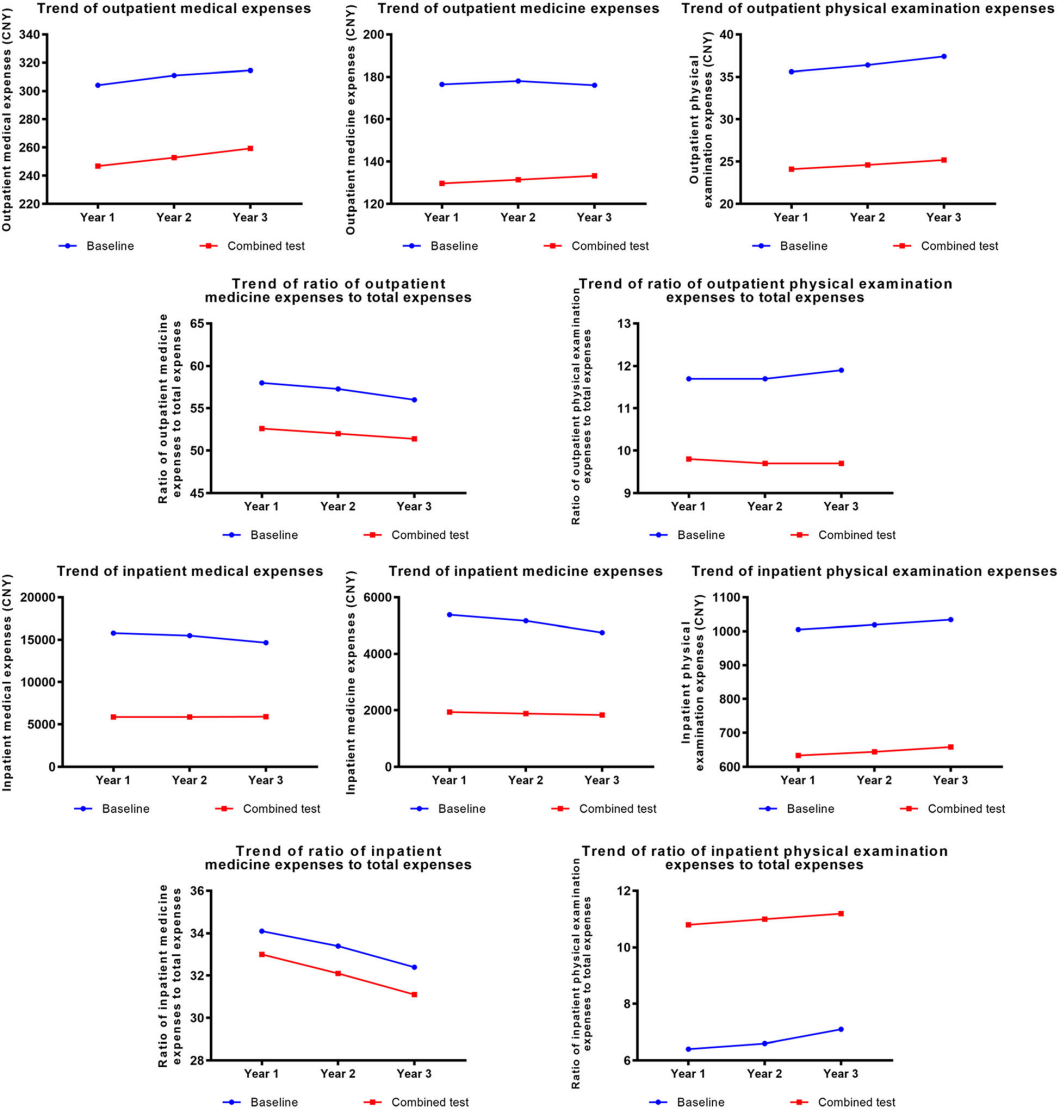
Results of combined policy intervention experiments

The intervention experiment results suggested that combined intervention could significantly reduce total medical expenses, medicine expenses and their proportions, physical examination expenses, and the proportion of outpatient physical examination expenses. The effect of combined intervention was better than that of independent experiments (Fig. 7).

## Discussion

At present, the medical expenses charged by public hospitals are unreasonable and keep increasing. The proportion of both medicine expenses and physical examination expenses to total medical expenses are also unreasonable. The intervention experiments conducted suggest that this could be attributed to patients’ unreasonable medical-seeking preferences, doctors’ weak public welfare incentives, and the government’s inadequate financial investment. Therefore, to control the unreasonable growth of medical expenses with the best effect, our findings indicated that interventions conducted simultaneously on suppliers, demanders, and the government, achieve better results when compared with isolated interventions on these agents.

First, expanding the degree of promotion and application of the community first-visit system could guide patients into community health service centers by rationalizing their medical-seeking preferences. It could thus achieve a reasonable diversion of patients, and effectively reduce outpatient and inpatient medical expenses. However, the promotion and application of the community first-visit system should be appropriate—expanding to 1.5 times the current degree was the maximum degree of effectiveness. In addition, considering the feasibility of this policy, it must be pointed out that the community health service centers have yet to play a gate-keeping role [40, 49, 50]. The main cause of the poor performance of the community first-visit system was human resource issues. For example, there is a lack of qualified GPs in primary healthcare institutions, especially in rural areas [40, 51, 52]. The limited medical capabilities of GPs working in community health service centers also impeded the acceptance of the community first-visit system by patients [53]. Therefore, for the community first-visit system to play a successful role in decreasing medical expenses, it is necessary to improve the development of primary healthcare institutions in China, and, in particular, address human resources issues.

Second, increasing doctors’ income and reducing their workload could significantly restrict doctors’ over-prescription behaviors; this would decrease outpatient and inpatient medical expenses by reducing extra expenses due to over-prescription. It is worth noting that increasing doctors’ income and reducing their workload disproportionately could lead to doctors’ inefficiencies. When their income increased twofold and working hours reduced to 75% of the current level, it reached the most significant effect. However, it should be noted that it was not easy to increase doctors’ incomes and reduce workload simultaneously. In high-level healthcare institutions, doctors face excessive work intensity because of too many patients; progressively more sudden deaths of Chinese doctors were reported globally in recent years [54, 55]. However, in primary healthcare institutions, doctors’ incomes kept growing with the increased investment from the government, and they did not suffer from excessive workload because of the current medical-seeking preferences for high-level hospitals. Therefore, before alleviating doctors’ workload, a method for shifting patient flow to lower-level hospitals needs to be determined. Apart from keeping the appropriate incomes and workload for doctors in these institutions, the more important issue to be addressed was how to accelerate the motivations of these doctors to be more innovative and improve their professional abilities, which would attract more patients to lower-level hospitals [15]. As a result, with an appropriate volume of patients in both higher- and lower-level hospitals, doctors would have more motivation to increase their professional standing and improve their quality of care, which was supported by an interview study [37].

Third, improving the government’s financial investment could guide public hospitals to strengthen their commitment to public welfare responsibilities, thereby reducing medical expenses effectively. However, the program had no significant effect on regulating the proportions of outpatient medicine and physical examination expenses. When investment into community health service centers increased to 175% of the current level, it achieved the best controlling effect. However, the threshold for the other three types of medical institutions was not reached, which suggests that the government’s financial investment into tertiary comprehensive, specialized, and district hospitals was extremely insufficient with much room for improvement. Since the implementation of China Health Reform in 2009, the Chinese government has continually increased the investment in public hospitals [56, 57]. However, with the reform gradually eliminating profits from drugs and medical instruments, the Chinese government should think more about how to compensate such profits to support the development of public hospitals [57].

Fourth, simultaneously rationalizing the patients’ medical-seeking preferences, increasing doctors’ income and reducing their workload, and increasing government financial investment achieved the best results for controlling the unreasonable growth of medical expenses. Policy makers should comprehensively examine the combined effects of various agents on medical expenses when formulating policy proposals. In addition, the feasibility, potential problems, and cost of such policies mentioned above should also be given full consideration before implementation.

This study had several limitations. First, due to the lack of complete statistics relating to medical expenses, the medical expense data in the model were measured according to the *China Health and Family Planning Statistical Yearbook*, consultation of medical staff in Shanghai public hospitals, and related experts. Although the extracted data performed well in verifying the model against the real system, it needs to be refined further in future research. Second, due to the complex simulation of the population’s mobility and the impact of disease transmission, this model assumed a statistically static populations distribution. Although the reasonable assumption could be understood, this assumption is far from the real world. Thus, it still needs to be revised in the next step. Third, although the “Opinions of the General Office of the State Council on Strengthening the Performance Appraisal of Tertiary Public Hospitals,” issued on January 30, 2019, noted that rational medicine use should be employed as the assessment indicator, rather than solely using the proportion of medicine expenses to total medical expenses, a reasonable indicator system has not yet been demonstrated. Therefore, this study used the proportion of medicine expenses to total medical expenses as an indicator to determine whether the medicine expenses were reasonable. A follow-up study should further discuss the rationality of medicine expenses. Fourth, although it was a reasonable simplification to some extent that the annual growth rate for doctors’ incomes in different types of hospitals did not differ based on previous national research and existing studies, it was a limitation that the differences among tertiary, specialized, district, and community-level hospitals were unspecified, especially the specialty of community health service centers. Therefore, further research on the different annual growth rates for doctors’ incomes in different types of hospitals and subsequent optimization of the model should be conducted.

## Conclusions

In conclusion, the current medical expenses of public hospitals continue to show a trend of unreasonable growth. This phenomenon of the unreasonable growth of expenses charged by public hospitals was affected mainly by patients’ unreasonable medical-seeking preferences, doctors’ weak public welfare motivation, and a lack of government public welfare guidance. Controlling the growth of these expenses can be mitigated effectively by comprehensively implementation of the community first-visit system, increasing doctors’ income and reducing their workload, and increasing government financial subsidies. However, the feasibility of these policies, underlying problems, and cost of such policies should be carefully examined before implementation.

## Supplementary information

**Additional file 1 Table S1.** Initial input values of parameters.

**Additional file 2 Table S2.** Assumptions of model.

## Data Availability

The datasets used and/or analyzed during the current study are available from the corresponding author on reasonable request.

## Abbreviation

MAS: Multi-agent system
