# Supplementary material for: Control of unreasonable growth of medical expenses in public hospitals in Shanghai, China: a multi-agent system model
Source: BMC Health Serv Res. 2020 Jun 3;20:490. doi: 10.1186/s12913-020-05309-z (PMC7268700; doi:10.1186/s12913-020-05309-z)
Supplement: Supplementary file 1 — Additional file 1 Table S1. Initial input values of parameters. [file 12913_2020_5309_MOESM1_ESM.docx]

**Table S1 Initial input values of parameters**

| Gender | Proportion |  |  |  |
| --- | --- | --- | --- | --- |
| Male | 0.515 |  |  |  |
| Female | 0.485 |  |  |  |
| Age (year) | Proportion |  |  |  |
| 0-4 | 0.0345 |  |  |  |
| 5-9 | 0.0275 |  |  |  |
| 10-14 | 0.0242 |  |  |  |
| 15-19 | 0.0487 |  |  |  |
| 20-24 | 0.1138 |  |  |  |
| 25-29 | 0.1117 |  |  |  |
| 30-34 | 0.0924 |  |  |  |
| 35-39 | 0.0835 |  |  |  |
| 40-44 | 0.0816 |  |  |  |
| 45-49 | 0.0782 |  |  |  |
| 50-54 | 0.0783 |  |  |  |
| 55-59 | 0.0749 |  |  |  |
| 60-64 | 0.0495 |  |  |  |
| 65-110 | 0.1012 |  |  |  |
| Occupation | Proportion |  |  |  |
| College students | 0.043 |  |  |  |
| Middle school students, children, infants | 0.127 |  |  |  |
| Retirement | 0.236 |  |  |  |
| Agriculture, forestry, animal husbandry and fishery | 0.0174636 |  |  |  |
| Mining industry | 0.0001782 |  |  |  |
| Manufacturing | 0.2103354 |  |  |  |
| Production and supply of electricity, gas and water | 0.0046926 |  |  |  |
| Construction industry | 0.0374814 |  |  |  |
| Transport, transportation, storage and postal services | 0.0447282 |  |  |  |
| Information transmission, computer services and software industry | 0.0131868 |  |  |  |
| Wholesale and retail trade | 0.092664 |  |  |  |
| Accommodation and catering | 0.0289278 |  |  |  |
| Financial industry | 0.0131274 |  |  |  |
| Real estate | 0.0182952 |  |  |  |
| Leasing and business services | 0.0223344 |  |  |  |
| Scientific research, technical services and geological prospecting | 0.008019 |  |  |  |
| Water, environment and public facilities management | 0.0076626 |  |  |  |
| Resident services and other services | 0.021681 |  |  |  |
| Education | 0.016929 |  |  |  |
| Health, social security and social welfare | 0.01188 |  |  |  |
| Culture, sports and entertainment | 0.0072468 |  |  |  |
| Public management and social organization | 0.0171072 |  |  |  |
| International organizations | 0.0000594 |  |  |  |
| Marital status | Proportion |  |  |  |
| Unmarried | 0.214 |  |  |  |
| Married | 0.723 |  |  |  |
| Divorced | 0.02 |  |  |  |
| Widowed | 0.043 |  |  |  |
| Monthly income (CNY) | Proportion |  |  |  |
| <2000 | 0.353 |  |  |  |
| 2000-4999 | 0.483 |  |  |  |
| 5000-7999 | 0.11 |  |  |  |
| ≥8000 | 0.055 |  |  |  |
| Education level | Proportion |  |  |  |
| Illiterate, semi-illiterate | 0.0315 |  |  |  |
| Primary school | 0.1413 |  |  |  |
| Junior high school | 0.3806 |  |  |  |
| High school, secondary vocational school | 0.2184 |  |  |  |
| Junior college | 0.1004 |  |  |  |
| Undergraduate | 0.1087 |  |  |  |
| Postgraduate | 0.0191 |  |  |  |
| Medical insurance | Proportion |  |  |  |
| Urban Employee Basic Medical Insurance-retirement (≥ 70 years old) | 0.0653 |  |  |  |
| Urban Employee Basic Medical Insurance-retirement (≤69 years old) | 0.1597 |  |  |  |
| Urban Employee Basic Medical Insurance-employee (≥45 years old) | 0.1571 |  |  |  |
| Urban Employee Basic Medical Insurance-employee (≤44 years old) | 0.483 |  |  |  |
| Urban and Rural Resident Basic Medical Insurance-residents (≥70 years old) | 0.0073789 |  |  |  |
| Urban and Rural Resident Basic Medical Insurance-residents (60-69 years old) | 0.0096502 |  |  |  |
| Urban and Rural Resident Basic Medical Insurance-residents (19-59 years old) | 0.0807272 |  |  |  |
| Urban and Rural Resident Basic Medical Insurance-undergraduate students | 0.004859 |  |  |  |
| Urban and Rural Resident Basic Medical Insurance-middle school students, children and infants | 0.014351 |  |  |  |
| Without medical insurance | 0.018 |  |  |  |
| Administrative district | Number of population (10,000) |  |  |  |
| Pudong New District | 547.49 |  |  |  |
| Huangpu District | 65.86 |  |  |  |
| Xuhui District | 108.91 |  |  |  |
| Changning District | 69.11 |  |  |  |
| Jing'an District | 23.69 |  |  |  |
| Putuo Dstrict | 128.80 |  |  |  |
| Zhabei District | 83.71 |  |  |  |
| Hongkou District | 80.94 |  |  |  |
| Yangpu District | 131.52 |  |  |  |
| Minhang District | 253.79 |  |  |  |
| Baoshan District | 202.29 |  |  |  |
| Jiading District | 156.80 |  |  |  |
| Jinshan District | 79.80 |  |  |  |
| Songjiang District | 176.02 |  |  |  |
| Qingpu District | 120.91 |  |  |  |
| Fengxian District | 115.99 |  |  |  |
| Chongming District | 69.64 |  |  |  |
| Disease | Two-week prevalence rate (‰) |  |  |  |
| Infectious disease | 0.8 |  |  |  |
| Malignant tumor | 3 |  |  |  |
| Benign tumor | 0.6 |  |  |  |
| Endocrine, nutritional and metabolic diseases | 52.1 |  |  |  |
| Of which: Diabetes | 47.5 |  |  |  |
| Blood diseases and hematopoietic diseases | 0.5 |  |  |  |
| Mental illness | 2.1 |  |  |  |
| Neuropathy | 3.5 |  |  |  |
| Eye and appendage diseases | 1.9 |  |  |  |
| Ear and mastoid disorders | 0.4 |  |  |  |
| Circulatory system diseases | 177.2 |  |  |  |
| Of which: Heart disease | 14.7 |  |  |  |
| Hypertension | 153.8 |  |  |  |
| Cerebrovascular disease | 6.4 |  |  |  |
| Respiratory diseases | 35.1 |  |  |  |
| Of which: Acute upper respiratory tract infection | 28.1 |  |  |  |
| Pneumonia | 0.6 |  |  |  |
| Bronchitis | 2.1 |  |  |  |
| Digestive system diseases | 13.7 |  |  |  |
| Of which: Acute gastritis | 6.2 |  |  |  |
| Cirrhosis | 0.4 |  |  |  |
| Gallbladder disease | 1.5 |  |  |  |
| Genitourinary diseases | 5.8 |  |  |  |
| Pregnancy, childbirth diseases and puerperal complications | 0.1 |  |  |  |
| Skin subcutaneous tissue diseases | 2.1 |  |  |  |
| Muscle and bone connective tissue diseases | 15.3 |  |  |  |
| Of which: Rheumatoid arthritis | 2.7 |  |  |  |
| Birth defects | 0.2 |  |  |  |
| Injury and poisoning | 4.5 |  |  |  |
| Hospital type | Hospital name (take 3 hospitals of each type as examples) | Latitude | Longitude |  |
| Tertiary hospital | Changzheng Hospital | N31°14′4.64″ | E121°27′45.99″ |  |
| Tertiary hospital | Renji Hospital | N31°12′34.40″ | E121°31′7.47″ |  |
| Tertiary hospital | Zhongshan Hospital | N31°11′56.20″ | E121°26′57.90″ |  |
| Specialized hospital | Shanghai Children's Hospital | N31°13′43.39″ | E121°22′53.83″ |  |
| Specialized hospital | Obstetrics & Gynecology Hospital | N31°12′50.98″ | E121°28′44.13″ |  |
| Specialized hospital | Shanghai Chest Hospital | N31°11′58.30″ | E121°25′14.07″ |  |
| District hospital | Shanghai East Hospital | N31°14′23.10″ | E121°30′28.86″ |  |
| District hospital | Shanghai Putuo Hospital | N31°14′49.54″ | E121°26′12.50″ |  |
| District hospital | Shanghai Post and Telcommunication Hospital | N31°13′13.86″ | E121°26′56.82″ |  |
| Community health service center | Changjiang Community Health Service Center | N31°20′31.22″ | E121°26′24.36″ |  |
| Community health service center | Sitang Community Health Service Center | N31°20′20.03″ | E121°27′14.45″ |  |
| Community health service center | Youyi Community Health Service Center | N31°24′12.97″ | E121°29′27.62″ |  |
| Professional title-doctors | Monthly income (CNY) | Proportion |  |  |
| Junior-Tertiary hospital | 6000 | 0.498 |  |  |
| Intermediate-Tertiary hospital | 6600 | 0.262 |  |  |
| Vice senior-Tertiary hospital | 7700 | 0.128 |  |  |
| Senior-Tertiary hospital | 10000 | 0.114 |  |  |
| Junior-Specialized hospital | 5800 | 0.723 |  |  |
| Intermediate-Specialized hospital | 6500 | 0.235 |  |  |
| Vice senior-Specialized hospital | 7500 | 0.036 |  |  |
| Senior-Specialized hospital | 9800 | 0.006 |  |  |
| Junior-District hospital | 5500 | 0.641 |  |  |
| Intermediate-District hospital | 6000 | 0.29 |  |  |
| Vice senior-District hospital | 7000 | 0.058 |  |  |
| Senior-District hospital | 9000 | 0.011 |  |  |
| Junior-Community Health Service Center | 4500 | 0.713 |  |  |
| Intermediate-Community Health Service Center | 5500 | 0.211 |  |  |
| Vice senior-Community Health Service Center | 6500 | 0.06 |  |  |
| Senior-Community Health Service Center | 8000 | 0.016 |  |  |
| Hospital type | Government investment proportion | Annual growth rate | Weekly working hour (hours) | Annual growth rate for doctors' incomes |
| Tertiary hospital | 0.062 | -0.00702 | 51.05 | 0.1104 |
| Specialized hospital | 0.0929 | -0.0314 | 51.09 | 0.1104 |
| District hospital | 0.0969 | 0.006825 | 51.13 | 0.1104 |
| Community health service center | 0.1516 | 0.094484 | 48.24 | 0.1104 |
