# Supplementary material for: Control of unreasonable growth of medical expenses in public hospitals in Shanghai, China: a multi-agent system model
Source: BMC Health Serv Res. 2020 Jun 3;20:490. doi: 10.1186/s12913-020-05309-z (PMC7268700; doi:10.1186/s12913-020-05309-z)
Supplement: Supplementary file 2 — Additional file 2 Table S2. Assumptions of model. [file 12913_2020_5309_MOESM2_ESM.docx]

**Table S2 Assumptions of model**

| **No.** | **Behavioral Assumption** | **Targeted agent** | **Detailed description** |
| --- | --- | --- | --- |
| 1 | Patient generation | Patients | (1) Classify diseases into 30 types according to China Health and Family Planning Statistical Yearbook;  (2) Be calculated by two-week prevalence rates of the 30 types of diseases;  (3) Regardless of the population’s mobility and the impacts of disease transmission. |
| 2 | Doctors' behaviors | (1) Targeted agent: doctors;  (2) Related agent: medical institutions, government | (1) Medical expenses are affected by doctors' over-prescription behavior;  (2) Ove-prescription is influenced by doctors' incomes and workload;  (3) Annual growth rates for doctors' incomes in different types of hospitals are assumed as the same rate. |
| 3 | Patients' medical-seeking behaviors | (1) Targeted agent: patients;  (2) Related agents: government, medical insurance agencies. | (1) The community first-visit system affects patients' medical-seeking preferences;  (2) The different reimbursement proportions of medical insurance in different levels of healthcare institutions affect patients' medical-seeking preferences. |
